# The publication fate of abstracts awarded prizes at European Society of Paediatric Radiology annual scientific meetings

**DOI:** 10.1007/s00247-024-06152-8

**Published:** 2025-01-22

**Authors:** Michael Paddock, Parasdeep S. Bains, Ola Kvist, Savvas Andronikou, Stephanie Franchi-Abella, Rick R. van Rijn, Owen J. Arthurs, Amaka C. Offiah, ESPR Publications Committee (consortium)

**Affiliations:** 1 SKG Radiology, Subiaco, Australia; 2 School of Medicine, The University of Notre Dame Australia, Fremantle, Australia; 3 School of Medicine and Population Health, University of Sheffield, Sheffield, UK; 4 Columbia University Irving Medical Center, New York, USA; 5 Department of Women’s and Children’s Health, Karolinska Institutet, Stockholm, Sweden; 6 Department of Radiology, Children’s Hospital of Philadelphia, Philadelphia, USA; 7 Perelman School of Medicine, University of Pennsylvania, Philadelphia, USA; 8 Paediatric Radiology Department, Bicêtre Hospital, Le Kremlin-Bicêtre, France; 9 Department of Radiology and Nuclear Medicine, Amsterdam UMC, University of Amsterdam, Amsterdam, Netherlands; 10 Department of Radiology, Great Ormond Street Hospital for Children, NHS Foundation Trust, London, UK; 11 Institute of Child Health, University College London, London, UK; 12 Department of Radiology, Sheffield Children’s NHS Foundation Trust, Sheffield, UK; 13 European Society of Paediatric Radiology, Paris, France

**Keywords:** Abstracts, Conferences and congresses, Medical societies, Publications, Radiology, Paediatric

## Abstract

**Background:**

The European Society of Paediatric Radiology (ESPR) awards prizes for outstanding work presented at their annual scientific meetings. The proportion of ESPR prize-winning abstracts to journal publications is not known. Contextualising abstract-to-publication proportions by evaluating publication experience can yield valuable insights and actionable outcomes to support researchers in overcoming barriers to journal publication.

**Objective:**

To assess the abstract-to-publication proportion of prize-winning ESPR abstracts and prize-winning authors’ experience of publishing in *Pediatric Radiology*, the affiliated journal of the ESPR and other specialist international paediatric radiology societies.

**Materials and methods:**

PubMed was searched for titles of ESPR prize-winning abstracts from 1977 (the year of first award) up to and including 2021, where the presenter was either first or co-author, and the article was published 2 years before or after the presentation year. If not found, a general internet search was performed. Titles of all retrieved articles were evaluated for inclusion. A survey was distributed to all ESPR prize winners to better understand their experiences around journal submission.

**Results:**

Over 44 years, 108 prizes were awarded. The prize-winning abstract-to-publication proportion was significantly higher (59.3%, OR=2.10, *P*=0.012) than the recently published pediatric radiology “abstract to publication rate” (41.9% from 2013–2016). Moreover, prize winners were more than twice as likely than to achieve journal publication (OR=2.10), and as first author (OR=1.33). The majority of awardees published their work as first author (52/64, 81.3%): the first-author abstract-to-publication proportion was not significantly higher than the paediatric radiology “abstract-to-publication rate” (48.1%, OR=0.33,* P*=0.330). Sixty-four survey responses were received (59.3%, out of a total 108 awarded prizes). Just over 20% of prize-winning work was published in *Pediatric Radiology*, with 41.5% of respondents reporting a good to excellent submission experience.

**Conclusion:**

Prize-winning and first-author abstract-to-publication proportions are higher for ESPR-awarded abstracts than the most recently reported paediatric radiology “abstract-to-publication rate”, suggesting that prizes are either awarded to work most likely to be published or that being awarded a prize encourages publication. Given that just over 40% of prize-winning abstracts remain unpublished, the ESPR should do more to support and encourage all authors to publish their work.

**Supplementary Information:**

The online version contains supplementary material available at 10.1007/s00247-024-06152-8.

## Introduction

The European Society of Paediatric Radiology (ESPR) held its first annual scientific meeting in 1964 in Paris, France. Every 5 years, a conjoint International Pediatric Radiology (IPR) congress is hosted by the ESPR and the Society for Pediatric Radiology (SPR), replacing their individual annual meetings. This first occurred in 1987 in Toronto, Canada [1]. *Pediatric Radiology* is the official journal for multiple international paediatric radiology societies, including the ESPR and SPR.

From presented work at these meetings, the ESPR bestows awards to “those who have enhanced the quality of paediatric radiology through innovation and scientific excellence”, the purpose of which is to “provide recognition for excellence in teaching and research and to promote [an] academic approach in paediatric radiology” [2]. The Jacques Lefèbvre Award was the first prize conferred at the 1977 annual scientific meeting in Lucerne, Switzerland. At the time of writing, there are six awards (and one research grant) that can be awarded to radiologists working in European institutions which can each be received only once per individual [2]. The rationale and criteria for each award are found on the ESPR website [2] and are summarised in Supplementary Material 1.

Eligible abstracts for consideration of ESPR prizes are judged on a combination of factors, including scientific content (study design, methodology and results); innovation and clinical utility; and clarity of presentation [2]. Since 2012, awardees have been chosen by the appointed scientific committee (of eight to ten members) during the respective annual scientific meeting of that year, led by the ESPR Research Committee Chair [2]. Prior to 2012, awardees were chosen by the appointed scientific committee led by the congress chairperson of that respective year (J. Tuschel, personal communication, 1 February 2022). No panel member scores work which is their own or which has been performed at their institution. Following the presentation of all abstracts, the independent scoring is reviewed at a consensus meeting and a final decision is made.

Subsequent journal publication of presented conference abstracts is considered a barometer of access to, and wider availability of, speciality publications and knowledge. The conversion of abstracts to journal publications may also be a gross indicator of concordance, or lack thereof, between scientific committee evaluation (which may evaluate preliminary data and conclusions) and the separate journal peer review process (appreciating the differing speed and depth of these processes).

In 1999, it was reported that “only approximately 30% of articles presented at SPR are published” [3]. In 2016, the conversion of orally presented abstracts at SPR, ESPR and IPR meetings to journal publication reportedly rose to nearly 42% [4]. A study in 2022 reported that while paediatric radiology “abstract to publication rates” within 12 months of abstract presentation increased from 29% (from 2010–2012) to 41.9% (from 2013–2016), more than half of assessed oral presentations at ESPR, SPR and IPR conferences remained unpublished [5]. Evaluations of other radiology congresses have demonstrated similar findings but which only assessed the conversion of orally presented abstracts to journal publication and not prize-winning work: 33% (Radiological Society of North America 1995, published between 1996–2000, *n* = 1,897) [6]; 39.5% (European Society of Gastrointestinal and Abdominal Radiology [ESGAR] 2000 and 2001, published between 2000–2004, *n* = 276) [7]; 40% (French Society of Radiology 2008–2010, published between 2008–2021, *n* = 744) [8]; 47% (European Congress of Radiology [ECR] 2000, published between 2000–2004, *n* = 1,020) [9]; and 51.8% (ECR 2010, published between 2010–2015, *n* = 869) [10].

Evaluating the publication experience of awardees is highly relevant and offers valuable insights into the barriers researchers face in converting high-quality, prize-winning abstracts to full journal publications. Moreover, contextualising abstract-to-publication proportions can identify factors influencing submission decisions, such as perceived journal fit, as well as facilitating benchmarking against other specialist society congresses and their affiliated journals, both within and outside radiology. Ultimately, this aligns with the goal of encouraging the dissemination of high-quality and impactful research.

We sought to assess the proportion of subsequent publication of prize-winning abstracts presented at ESPR annual scientific meetings from 1977–2022 to publication in *Pediatric Radiology*, and which factors impact journal submission choice.

## Methodology

The year, name of awardee, country and title of abstracts awarded prizes from 1977–2021 were retrieved from the ESPR website [2]. The following awards were assessed: Jacques Lefèbvre Award; Scientific and Educational Poster Awards; Young Researcher Award; President’s Award; and the Innovation Award. The Other Award (awarded for outstanding presentations if felt justified by the ESPR Research Committee) was not assessed as it fell outside the time frame of this review, with only one award documented in 2022. The Guy Sebag research grant was not included as it is not an ESPR prize and is funded by a separate grant panel.

Manual searches of article titles were performed using the PubMed database (https://pubmed.ncbi.nlm.nih.gov) by study authors using abstract titles from the ESPR website. If an awarded abstract title was not identified, a general internet search was performed to find articles with the same/similar title and/or published in a non-PubMed indexed journal. If published in any journal, the following data were retrieved: name; year; title; country; first author; co-authors; PubMed identifier (PMID, if applicable); and the title and PMID of any related article(s).

Published articles were included only when: the title was the same as (or a close variation of) that published on the ESPR website within 2 years either side of the presentation year; the abstract presenter was either the first or co-author; the article was published in English, as the official language of ESPR annual scientific meetings. Where there was a variation in title, the titles and authors of any retrieved related articles were evaluated to ensure that the correct publication was included. Given our imposed 2-year publication time frame, only abstracts presented up to and including the 2021 ESPR annual scientific meeting were captured. No awards were made in 2020 as the 56^th^ ESPR annual scientific meeting, due to be held in Marseille, France, was cancelled due to the coronavirus disease 2019 (SARS-CoV-2) pandemic.

The following outcomes were assessed:

- Abstract-to-publication proportion – the number of ESPR prize-winning recipients who subsequently published their prize-winning work, divided by the total number of ESPR prize winners
- First-author abstract-to-publication proportion – the number of ESPR prize-winning recipients who subsequently published their prize-winning work as first author, divided by the total number of ESPR prize winners

The outcomes were compared to the most recently published paediatric radiology “abstract to publication rate” defined as the proportion of abstracts presented at ESPR, SPR and IPR conferences that subsequently published in paediatric radiology journals within 12 months of conference presentation [5].

A Qualtrics survey (Supplementary Material 2) was created and distributed to individual ESPR prize winners via email, as well as advertised to ESPR members via the ESPR newsletter. If an individual was awarded more than one prize, they were asked to complete separate entries for each award. The survey was open from 19 January 2024 to 7 April 2024.

Neither ethical nor institutional board approval was required for this retrospective bibliometric review and survey. Descriptive statistics and two-tailed Fisher’s exact tests were employed (IBM SPSS Statistics for Windows, version 29.0.1.0, IBM Corporation, Armonk, NY). Statistical significance was set at *P* < 0.05.

## Results

Since 1977, 108 ESPR prizes have been awarded. The prize-winning abstract-to-publication proportion was significantly higher (59.3%, OR = 2.10, *P* = 0.012) than the most recently reported paediatric radiology “abstract to publication rate” (41.9%). The majority of awardees published their work as first author (52/64, 81.3%). The first-author abstract-to-publication proportion was higher than the paediatric radiology “abstract to publication rate” but not significantly so (48.1%, OR = 1.33,* P* = 0.330). Approximately one-third of published abstracts (22/65, 33.8%) were published in *Pediatric Radiology*. The data for each award are displayed in Table 1.

**Table 1:** Summary of the prizes awarded at European Society of Paediatric Radiology annual scientific meetings since 1977, by the number of times awarded and the abstract-to-publication proportion for each award and for first author

Prize	Year first awarded	Monetary value (€)	Reason for award	Years not awarded^a^	Number of times awarded	Abstract-to-publication proportion % (published/awarded)	First-author abstract-to-publication proportion % (published/awarded)	Number published in *Pediatric Radiology* % (published/all published)
Jacques Lefèbvre Award	1977	800	Best scientific research paper	2020	44	77.3 (34/44)	63.6 (28/44)	17.2 (11/64)
Poster Awards (two)	1994	300 (each)	Best scientific poster	1996, 1999, 2020	25	40.0 (10/25)	28.0 (7/25)	4.7 (3/64)
			Best educational poster		6	16.7 (1/6)	16.7 (1/6)	1.6 (1/64)
Young Researcher Award	2003	500	Outstanding contributions by young scientists aged less than 35 years for the best poster or scientific paper	2009, 2020	17	52.9 (9/17)	47.1 (8/17)	4.7 (3/64)
President’s Award	2004	500	Best national oral or poster presentation	2006, 2019, 2020	15	66.7 (10/15)	46.7 (7/15)	6.3 (4/64)
Innovation Award^b^	2021	500	Most innovative oral or poster presentation	2012 to 2020	1	100.0 (1/1)	100.0 (1/1)	0.0
Total					108	59.3 (64/108)	48.1 (52/108)	22/64 (34.4)

^a^No awards were made in 2020 as the 56th ESPR annual scientific meeting in Marseille, France, was cancelled due to the coronavirus disease 2019 (SARS-CoV-2) pandemic

^b^The Innovation Award is an ad hoc award with only one documented award in 2021

The outcome of any given award is compared to outcomes of all ESPR awards. The Jacques Lefèbvre Award had the highest prize-winning (77.3%, OR = 18.36, *P* < 0.001) and first-author (63.6%, OR = 16.92, *P* < 0.001) abstract-to-publication proportions. Of the remaining ESPR awards, there were lower prize-winning and first-author abstract-to-publication proportions for the Young Researcher Award (52.9%, OR = 0.74, *P* = 0.599; and 47.1%, OR = 0.95, *P* = 1.000, respectively) and the President’s Award (66.7%, OR = 1.44, *P* = 0.584; and 46.7%, OR = 0.93, *P* = 1.000, respectively).

Sixty-four survey responses were received (59.3%, out of a total 108 prizes awarded); however, not all respondents answered every question. A summary of survey responses is presented in Table 2.

**Table 2:** Summary of survey responses

Question	Yes (%)	No (%)
Was your research published in *Pediatric Radiology*?	27/64 (42.2)	37/64 (57.8)
Did you submit your awarded research to *Pediatric Radiology*?	21/40 (52.5)	19/40 (47.5)
Was *Pediatric Radiology* your first choice journal in which to publish your research?	21/26 (80.8)	5/26 (19.2)
Was your research published in your first choice journal?	15/35 (42.9)	20/35 (57.1)

Sixty-four survey responses were received (59.3%, out of a total 108 awarded prizes). Not all respondents answered every question

Of authors who published in journals other than *Pediatric Radiology* (37/64, 57.8%), less than half of respondents (15/35, 42.9%) indicated that this journal was their first choice journal. Wide readership was cited as the most important factor for first choice journal selection by most respondents (28/46, 60.9%), followed by journal impact factor (18/46, 39.1%).

## Discussion

Our study demonstrates that over half (59.3%) of all presented abstracts awarded prizes at ESPR annual scientific meetings since 1977 have been published. This is a significantly higher proportion than the recently reported publication proportion for abstracts presented at ESPR, SPR and IPR conferences (41.9%), with ESPR prize-winning work more than twice as likely to achieve subsequent journal publication (OR = 2.10). There may be several factors which contribute to this: the superior quality of the work as reflected by the award of a prize, [11, 12] the enhanced recognition associated with the award of a prize which may encourage an author to publish their work, or that work most likely to be achieve publication is awarded a prize. Despite the higher publication proportion of ESPR prize-winning work, it is discouraging that just over 40% of awarded work deemed to be of high scientific quality and import remains unpublished. The ESPR awards a total of €2,900 in prize money annually. As such, the society has a vested interest in encouraging and supporting presenters to convert their prize-winning abstracts into journal publications.

Excluding the sole *ad hoc* Innovation Award, the Jacques Lefèbvre Award, awarded to the best scientific research paper presented by an investigator under the age of 40 years, has the highest prize-winning and first-author abstract-to-publication proportions. Individuals awarded this prize are over 18 times more likely to achieve publication, and nearly 17 times more likely to do so as first author. Compared to the Jacques Lefèbvre Award, recipients of the Young Researcher and President Awards are less likely to achieve journal publication, but with no significant difference in the likelihood of publication as first-author. The lower likelihood of publication may reflect the variability in available resources (e.g. access to senior research colleagues, mentorship) or limitations in experience (this award is made to young scientists aged less than 35 years). While it is possible that senior radiologists may voluntarily step aside to encourage junior colleagues to take the lead in authorship, junior radiologists may decide to relinquish first-author responsibilities due to the (perceived or actual) increased workload while fulfilling their specialty training obligations alongside preparing for specialty/fellowship examinations, provided that the work is eventually published by a co-author.

Poster presentations are a valuable tool for sharing early-stage research and providing junior researchers with a platform to build confidence and develop academic skills in a less formal setting than oral presentations [13, 14]. While posters may be upgraded to oral presentations if deemed of sufficient quality, they are often perceived as “lesser” [15], reflecting a hierarchy that may discourage some from pursuing publication. Educational posters, typically of the “pictorial review” type rather than original scientific articles, face higher competition for journal publication, with only one prize-winning educational poster achieving first-author publication since 2015. This may stem from their less-developed content or perceived lower prestige, necessitating further refinement to meet journal submission standards for eventual publication.

The award of a prize may serve to not only satisfy individual personal and professional aspirations but may also enhance the motivation to achieve journal publication. Notwithstanding, if presented abstracts do not go on to achieve full publication, there risks preliminary, incomplete or invalid data being published in conference abstract issues of the journal: the same applies to full journal publications, the main difference being the rigour of peer review. A Cochrane systematic review reported that of work presented as posters at scientific meetings, only around half went onto be published as full articles in peer-reviewed journals [17], whereas work presented orally was more likely to achieve full publication (RR = 1.46; 95% CI 1.40–1.52). Our findings support this research.

The survey findings highlight the challenges and decision-making processes that authors face in journal submission. The results demonstrated that despite an even distribution between work published and not published in *Pediatric Radiology*, just less than half of respondents (19/40, 47.5%) did not submit their work for consideration to this journal. Factors that individuals may consider when selecting a journal in which to publish include journal reputation and quality, publication speed and journal impact factor [17]. The primary reason cited by our respondents for non-submission was the perception of their work to be of greater appropriateness and relevance to other specialty journals, suggesting that ESPR prize-winning work is being published outside of the society journal. The ESPR could actively encourage prize winners to submit their work to Special Issues of *Pediatric Radiology*. The scope of the journal could be further publicised and reiterated at ESPR annual scientific meetings. The ESPR newsletter, ESPR educational and taskforce webinars/events, and the European Course in Paediatric Radiology (ECPR) series are (possibly underutilised) opportunities to publicise and attract high-quality submissions.

There are several study limitations, in particular, the retrospective nature of this bibliometric review and survey, the latter being subject to non-response in terms of total number of respondents, attrition (not all respondents answered every question), and recall bias. The 2-year time frame employed in this study was felt to be sufficient to capture most journal publications resulting from preliminary or pilot work presented at annual scientific meetings but the inevitable time lag between the award of a prize and the subsequent publication of work may not have captured all available data. It is possible that not all relevant published work was identified through our searches. Moreover, non-publication could result from several factors including but not limited to loss of project support, changes in team interest and configuration, time constraints, non-submission, and non-acceptance of work. We did not measure the rejection rates of journal submission by prize category. Finally, only studies published in English were retrieved.

## Conclusion

The proportion of ESPR prize-winning abstracts that go on to achieve journal publication is significantly higher than the most recently published paediatric radiology “abstract to publication rate” and is more than twice as likely to achieve publication. However, it is disappointing that just over 40% of abstracts awarded prizes remain unpublished and only just over 20% of prize-winning work is published in *Pediatric Radiology*, the official journal of the ESPR. The award of congress prizes provides incentive to stimulate research within the paediatric radiology community across Europe. Publication of prize-winning work can serve to enhance the reputation of our specialty and provide a platform to encourage the next cohort of paediatric radiologists to take up the mantle of research.

## Supplementary Information

Below is the link to the electronic supplementary material.

Supplementary file1 (DOCX 17.3 KB)

Supplementary file2 (DOCX 26 KB)

## Data Availability

Data is provided within the manuscript or supplementary information files.
