# Supplementary material for: The publication fate of abstracts awarded prizes at European Society of Paediatric Radiology annual scientific meetings
Source: Pediatr Radiol. 2025 Jan 22;55(3):578–84. doi: 10.1007/s00247-024-06152-8 (PMC11882696; doi:10.1007/s00247-024-06152-8)
Supplement: Supplementary file 1 — Supplementary file1 (DOCX 17.3 KB) [file 247_2024_6152_MOESM1_ESM.docx]

**Supplementary Material 1**

**European Society of Paediatric Radiology Awards and Grants**

- **Jacques Lefèbvre Award** (since 1997) – for the best scientific research paper presented at the annual ESPR congress. The recipient should be under the age of 40 years and will be invited free-of-charge to present their paper at the next SPR/IPR meeting.
- **Educational and Scientific Poster Awards** (since 1994) – for the best poster presented in each category (case reports and pictorial reviews are considered for the educational poster prize). There are no age restraints. Separate scientific and education prizes were awarded from 2015 onwards.
- **Young Researcher Award** (since 2003) – for outstanding contributions to paediatric imaging by young scientists for the best poster or scientific paper. The recipient should be under the age of 35 years.
- **President’s Award** (since 2004) – given at the discretion of the sitting meeting president for the best national oral or poster presentation (awarded to a presenter from the country in which the annual scientific meeting is being held). There are no age constraints.
- **Innovation Award** (since 2021) – made for the most innovative oral or poster presentation, whether scientific or educational. There are no age constraints.
- **Other Award** (*ad hoc*, since 2022)) – additional awards/prizes made for outstanding presentations, if felt justified by the ESPR Research Committee.
- **Guy Sebag Grant** (since 2017) – made for clinical, basic or translational research in paediatric radiology, aiming at stimulating new research initiatives rather than funding mature research programs, including pilot studies that will subsequently lead to larger studies in *Pediatric Radiology*.

European Society of Paediatric Radiology (2022) ESPR Awards and Grants. European Society of Paediatric Radiology. Available at <https://www.espr.org/about-espr/> Accessed 3 December 2024.
