# Supplementary material for: The publication fate of abstracts awarded prizes at European Society of Paediatric Radiology annual scientific meetings
Source: Pediatr Radiol. 2025 Jan 22;55(3):578–84. doi: 10.1007/s00247-024-06152-8 (PMC11882696; doi:10.1007/s00247-024-06152-8)
Supplement: Supplementary file 2 — Supplementary file2 (DOCX 26 KB) [file 247_2024_6152_MOESM2_ESM.docx]

Publication Committee Survey

Was your research published in Pediatric Radiology?

- Yes
- No

Was Pediatric Radiology your first-choice journal in which to publish your research?

- Yes
- No

Please provide the name of your first-choice journal for publication.

________________________________________________________________

Why was your research published in Pediatric radiology, rather than your first-choice journal?

________________________________________________________________

Was your research published in your first-choice journal?

- Yes
- No

Please provide the name of your first-choice journal for publication

________________________________________________________________

Please provide the name of the journal in which your manuscript was published?

________________________________________________________________

Why was your research published in its eventual journal rather that your first-choice journal?

________________________________________________________________

Was a prize awarded for a poster presentation or an oral (podium) presentation?

- Poster
- Oral (Podium)
- Other (please specify below) __________________________________________________

Why was your chosen journal your first-choice journal? (Choose up to three)

- Impact factor
- Wide readership
- Time of publication
- Quality of reviews
- Open Access (free or covered by institution or other)
- Other (please specify below) __________________________________________________

Did you ever submit your awarded research to Pediatric Radiology for Publication?

- Yes
- No

Why did you not submit your awarded research to Pediatric Radiology?

__________________________________________________

How was your experience submitting the awarded abstract to Pediatric Radiology for publication (if relevant)?

- Poor
- Fair
- Average
- Good
- Excellent
- Did not submit to Pediatric Radiology

How was your experience of your most recent Pediatric Radiology publication?

- Poor
- Fair
- Average
- Good
- Excellent

How was your experience of your most recent publication to another journal?

- Poor
- Fair
- Average
- Good
- Excellent
- I have not submitted manuscripts to other journals

In which journal was your most recent publication?

**Free text responses to Publication Committee Survey**

**Q4 - Why was your research published in *Pediatric Radiology*, rather than your first-choice journal?**

It was not published in Pediatric Radiology x 2

It got rejected

**Q6 - Why was your chosen journal your first-choice journal? (Choose up to three) - Selected Choice**

Other (Free text):

Prize ESPR congress

Not submitted

The results became two separate manuscript that were published at two different journals

Invited to this journal

Most relevant for this research

Best suited for the topic

**Q9 - Please provide the name of your first-choice journal for publication.**

Hepatology x 2

Not submitted to any journal

Bone and Joint Journal

Radiology

UOG and Clinical Genetics Journal

European Radiology x 2

**Q10 - Why was your research published in its eventual journal rather that your first-choice journal?**

Rejection by hepatology

Rejection by Pediatric Radiology

Not accepted in first choice journal x 3

Educational Poster, not published

Not published x 2

**Q11 - Please provide the name of the journal in which your manuscript was published?**

Journal of Pediatric Gastroenterology and Nutrition (JPGN) x 3

Cancers

Bone and Joint Journal

Not published x 2

Korean Journal of Radiology

**Q18 - Why did you not submit your awarded research to *Pediatric Radiology*?**

The article was not only radiologic but had a significant part dedicated to clinical presentation

Other coauthors had other wishes

Topic more relevant in another journal

Preferred another journal

Higher impact factor in European Radiology x 2

Submitted to another journal before award

Orthopedic journal better suited for the manuscript

Published in Annals of the Rheumatic Diseases to reach out to a larger group of clinicians

Invited to another journal

I do not remember x 2

**Q15 - In which journal was your most recent publication?**

Pediatric radiology x11

Prenatal Diagnosis

Diagnostic Interventional Imaging (DII) x2

Circulation

AJNR

Radiology x2

Pediatrics x2

Die Radiologie

European Journal of Radiology

AJR invited publication

European Radiology

Investigative Radiology

British Journal of Radiology

Academic Radiology

Japan J of Radiology

Spine

Prenatal Diagnosis

Clinical Radiology

RoFo Thieme

Radiology Case reports

Children

Fortschr Roentgenstr

Clinical Radiology

Nature
